# PTCSC3‐mediated glycolysis suppresses thyroid cancer progression via interfering with PGK1 degradation

**DOI:** 10.1111/jcmm.16806

**Published:** 2021-08-01

**Authors:** Bo Jiang, Yong Chen, Fada Xia, Xinying Li

**Affiliations:** ^1^ Department of General Surgery Xiangya Hospital Central South University Changsha China

**Keywords:** glycolysis, LncRNA, PGK1, PTCSC3, thyroid cancer

## Abstract

The Warburg effect (aerobic glycolysis), a hallmark of cancer, serves as a promising target for diagnosis and therapy. Growing evidence indicates that long non‐coding RNAs (lncRNAs) play an important role in aerobic glycolysis of various tumours. However, the correlation between lncRNAs and glycolysis in thyroid cancer cells is still poorly understood. In this study, we showed that lncRNA papillary thyroid cancer susceptibility candidate 3 (PTCSC3) was significantly downregulated in papillary thyroid carcinoma (PTC). Overexpression of PTCSC3 significantly inhibited the aerobic glycolysis and tumour growth of PTC cells. Consistently, PTCSC3 overexpression suppressed tumour progress in vivo. Mechanistically, PTCSC3 inhibits aerobic glycolysis and proliferation of PTC by directly interacting with PGK1, a key enzyme in glycolytic pathway. As a result, PTCSC3 performs its role in PTC development via PGK1 and may be a potential therapeutic target for PTC treatment.

## 1 INTRODUCTION

Thyroid cancer is the most common malignant tumour of endocrine system.^1^, ^2^ In recent years, the incidence of thyroid cancer continues to increase rapidly all over the world.^3^, ^4^ Particularly, papillary thyroid carcinoma (PTC) is the most common subtype of thyroid cancer, comprising 80% to 90% of all thyroid cancers.^5^ In general, well‐differentiated PTC is regarded as a fairly indolent tumour with long‐term survival rates >95%. However, there are certain variants of PTC that are more aggressive in nature with less favourable disease‐free survival and overall survival.^6^ These variants differ in histology, cytology, molecular markers, treatment strategies and outcomes. Moreover, the precise mechanism underlying pathogenesis of thyroid cancer is incompletely understood. Therefore, exploring pathogenetic mechanisms of thyroid cancer is urgently required to identify treatment modalities and therapeutic intervention for PTC.

Cancer cells exhibit a higher rate of glucose consumption and lactate production in the presence of oxygen than their normal differentiated counterparts.^7^ This phenomenon, which was first noted by Warburg in the 1920s and known as aerobic glycolysis or the Warburg effect, allows tumour cells to function like foetal cells and to use a large fraction of glucose metabolites to synthesize macromolecules (such as amino acids, phospholipids and nucleic acids), which support tumour cell growth. Aerobic glycolysis in solid tumour has been reported to contribute to the tumorigenesis in various malignancies, including non–small‐cell lung cancer and breast cancer. The aerobic glycolysis provided cancer cells the advantage of gaining sufficient biomass‐building materials for cell growth and proliferation and adapting highly oxidative microenvironment for better survival, thus become a promising therapeutic intervention for cancer.^8^, ^9^, ^10^

PGK1, a key enzyme in glycolytic pathway, reversibly catalyses the reaction of 1,3‐bispho‐spholgycerate (1,3‐BPG) and ADP into 3‐phosphoglycerate (3‐PG) and ATP.^11^ This catalysed reaction regulated by PGK1 is the first ATP‐generating step of glycolysis and is crucial for energy production and biosynthesis via the glycolytic pathway in most living cells.^12^ It is known that PGK1 is upregulated in a variety of malignant cancers, such as hepatocellular carcinoma,^13^ pancreatic ductal adenocarcinoma^14^ and lung cancer.^15^ However, the precise mechanisms of PGK1 regulating glycolysis and tumorigenesis in thyroid cancer are largely unknown.

Non‐coding RNAs, same as microRNA, circular RNA or long non‐coding RNA, are emerging as important regulators of gene expression.^16^ Long non‐coding RNAs are functionally defined as transcripts with a length of more than 200 nucleotides, which have no appreciable protein coding potential.^17^ Importantly, in recent years, it has become clear that LncRNAs play critical roles in diverse physiological and pathological processes.^18^, ^19^, ^20^ Biologically, the dysregulated expression of LncRNAs in a variety of cancers results in abnormal tumour proliferation, migration and apoptosis, contributing to tumour development and progression.^21^, ^22^, ^23^ For example, LINC02273 plays a crucial role in breast cancer metastasis by increasing AGR2 transcription.^24^ KRT19P3, which is downregulated in gastric cancer, suppresses tumour growth and metastasis through COPS7A‐mediated NF‐κB pathway.^25^ GBCDRlnc1 induces chemoresistance of gallbladder cancer cells via activating autophagy.^26^ Previous study indicated that long non‐coding RNA papillary thyroid carcinoma susceptibility candidate 3 (PTCSC3) is an anti‐tumour LncRNA,^27^ but the explicit mechanism still remains to be identified.

A large number of studies have shown that LncRNAs can interact with proteins to participate in various biological processes, such as chromatin remodelling, transcription regulation and RNA degradation.^28^, ^29^ For example, through binding with PRPF19 and PTPN11, LINC00673 strengthens the PRPF19–PTPN11 interaction, contributing to enhance PRPF19‐mediated ubiquitination and degradation of PTPN11 in pancreatic ductal adenocarcinoma.^30^ MetaLnc9 interacts with NONO and promotes the recruitment of NONO and CRTC2 to regulate its own expression.^31^

In this study, we observed that PTCSC3 is significantly downregulated in papillary thyroid cancer. Moreover, we further discovered that PTCSC3 inhibited thyroid cancer development by suppressing glycolysis. Mechanistically, PTCSC3 directly bonds to PGK1, modulates its ubiquitination degradation and eventually inhibits PTC progression. The tumour‐suppressive function of PTCSC3 and the underlying mechanisms were clarified.

## 2 METHODS AND MATERIALS

### 2.1 Ethics approval and consent to participate

This study was approved by the ethics committee of Xiangya Hospital, Central South University (CSU; Changsha, China). All patients were provided with written informed consent. All animal studies were approved by the Animal Ethics Committee and processed in accordance with the official recommendations of the Care and Use of Laboratory Animals of Xiangya Hospital, CSU.

### 2.2 Patients and tissue specimens

PTC tissues and adjacent normal tissues were gathered from patients who underwent surgical thyroid resection at the Department of Thyroid Surgery, Xiangya Hospital, CSU. Patients did not receive any local or systemic treatment before the thyroid resection operation. Clinicopathological TNM staging was judged according to the 8th thyroid cancer TNM classification criteria.

### 2.3 Cell lines and cell culture

Nthy‐ori 3‐1 (abbreviated as Nthy thereafter), TPC‐1 and BCPAP cells were purchased from the Chinese Academy of Science Cell Bank. For cell line authentication, short tandem repeat profiling was performed before the beginning of experiment. All cell lines were maintained in RPMI‐1640 (Gibco) media supplemented with10%FBS (Gibco) and 1% ampicillin/streptomycin (Gibco) and cultured at 5% CO2 and 37°C. All cell lines were passaged <10 times. Cells were treated with 2‐DG (2.5 mM), MG132 (10 μM), CHX (20 μg/ml) and oligomycin (0.1 mM) at indicated times, respectively.

### 2.4 RNA extraction and real‐time quantitative polymerase chain reaction

Total RNA was isolated from cells and frozen tissue specimens using TRIZOL reagent (Invitrogen) according to the manufacturer's protocol. cDNA was generated using PrimeScript Reverse Transcription kit (Takara). Quantitative real‐time PCR was carried out by SYBR Premix Ex Taq II (TaKaRa) using the ViiA 7 real‐time PCR system (Applied Biosystems) following the manufacturer's instructions. Relative expression levels were determined using the ΔCt method, and GAPDH was measured as an internal control. Each sample was examined in triplicates.

The qRT‐PCR primers were as follows: PTCSC3, forward: 5′‐ TCCAGGGGGATCGCATTTTT‐3′, reverse: 5′‐ GCCTTTGACCTGGTCTCTCC ‐3′; PGK1, forward: 5′‐CCACTGTGGCTTCTGGCATA‐3′, reverse: 5′‐ ATGAGAGCTTTGGTTCCCCG‐3′; GAPDH: forward: 5′‐ TGCACCACCAACTGCTTAGC ‐3′, reverse: 5′‐ GGCATGGACTGTGGTCATGAG‐3′.

### 2.5 Lentivirus transduction

The lentivirus encoding PTCSC3, control lentivirus and PGK1 were obtained from Vigene Biosciences (USA). Cells were transfected at 50% confluency with a final lentivirus multiplicity of infection (MOI) of 20. After lentivirus transfected, TPC‐1 and BCPAP cells were treated with 2 μg/ml puromycin to establish stable cell lines.

### 2.6 Cell counting kit‐8 and colony formation assays

The cell proliferation capacity was performed by cell counting kit‐8 (CCK8) (Yeasen Biotech Co., Ltd). Transfected TPC‐1 and BCPAP cells (3 × 103/well) were seeded in 96‐well plates. The absorbance was measured at 450 nm on 0, 24, 48, 72 and 96 h after the cell seeding.

Transfected TPC‐1 and BCPAP cells (200 cells/well) were seeded in 6‐well plates to evaluate the monolayer colony formation ability. The cells were cultured with RPMI‐1640 medium (Gibco) at 37°C for 1–2 weeks. Then, the adherent cells were washed three times with PBS and fixed with 4% paraformaldehyde for 30 min at room temperature and stained for 15 min with 0.5% crystal violet (Chengdu Kelong Chemical Co., Ltd.) at room temperature. Light microscopy was used to count the cell colonies (>50 cells per colony). All assays were conducted in triplicate.

### 2.7 Glucose uptake, lactate, ATP assays

Glucose Uptake Colorimetric Assay Kit (BioVision), Lactate Assay Kit (BioVision) and ATP Colorimetric Assay Kit (BioVision) were used to determine glucose uptake, levels of lactate and ATP, respectively, according to the manufacturer's protocols. 1 × 10^4^ cells were seeded into a 96‐well plate and incubated for 4 h, washed twice by PBS and starved in 100 µl 1640 without FBS overnight. Cells were washed three times with PBS and then incubated with 100 μl for Krebs‐Ringer‐Phosphate‐HEPES (KRPH) buffer containing 2% BSA for 40 min. Then, 10 μl of 10 mM 2‐DG was added and incubated for 20 min. Cells were lysed with 80 ml of extraction buffer and then frozen/thawed once and heated at 85°C for 40 min. Adding 10 ml of neutralization buffer neutralized the cell lysate. The sample was used for determination of glucose uptake. The glucose uptake was measured at 412 nm in a microplate reader.

For measurement of lactate production, 1 × 10^4^ cells were seeded into a 12‐well plate and cultured in 1640 containing 10% FBS overnight. Then, the media were removed and washed twice by PBS, and the cells were cultured in 1640 without FBS. After incubation for 1 h, the supernatant was collected for measurement of lactate production (BioVision). The reaction mixture was incubated for 30 min at room temperature. The lactate levels were measured at 450 nm in a microplate reader.

For measurement of the lactate levels of mouse tumour, 10 mg of tumour tissues were isolated and homogenized in the Assay Buffer. The samples were centrifuged, and the soluble fractions were measured. The lactate levels were measured as described above.

For ATP level analysis, cells were lysed in 100 μl ATP Assay Buffer. After deproteinizated by using Deproteinization Sample Preparation Kit (BioVision), the sample was collected for ATP determination. The reaction mixture was incubated for 30 min at room temperature, protected from light and measured at 570 nm in a microplate reader.

### 2.8 RNA pull‐down assay and mass spectrometry

RNA pull‐down assay was performed using Pierce Magnetic RNA‐Protein Pull‐down kit (Thermo Scientific) according to the manufacturer's instructions. Briefly, sense and antisense PTCSC3 were in vitro transcripted by T7 RNA polymerase using MEGAscript kit (Ambion) and labelled by Pierce RNA 3′ Desthiobiotinylation Kit (Thermo Scientific), then purified by phenol:chloroform mixture, added 1 μg biotin‐labelled RNA with 50 μl streptavidin magnetic beads for 30 min at room temperature with rotating and incubated with cell lysates overnight. The eluted proteins were detected by mass spectrometry and Western blot analysis.

### 2.9 RNA immunoprecipitation assay

RNA immunoprecipitation assays were performed using Magna RNA‐binding Protein Immunoprecipitation Kit (Millipore) according to the manufacturer's instructions. Cells were homogenized in100 μl RIP lysis buffer with 0.5 μl Protease inhibitor and 0.25 μl RNase inhibitor on ice for 5 min and then stored at −80°C. Magnetic beads were incubated with 5 μg Mouse anti‐PGK1 (Santa Cruz Biotechnology, sc‐130335) and 5 μg Mouse anti‐lgG (Millipore, CS200621) at room temperature for 30 min, respectively. RIP lysate and beads‐antibody complex were incubated with RIP immunoprecipitation buffer containing 35 μl of 0.5 M EDTA, 5 μl RNAse inhibitor and 860 μl RIP wash buffer at 4°C overnight. Co‐precipitated samples were digested by proteinase K buffer containing 117 μl RIP wash buffer, 15 μl of 10% SDS, 18 μl of 10 mg/ml proteinase K at 55°C for 30 min with shaking and then the immunoprecipitated RNAs were extracted and purified for utilizing. The enrichment was determined by qPCR and RT‐PCR with agarose gel electrophoresis analysis.

### 2.10 Western blot assay and immunoprecipitation

Cells were homogenized in RIPA buffer with Protease/Phosphatase Inhibitor Cocktail. Lysates were incubated on ice for 30 min and centrifugated at 14,000 rpm for 15 min. The supernatants were collected and measured by BCA kits and prepared for immunoblotting or immunoprecipitation with the interest antibodies. The proteins were separated by SDS‐PAGE and transferred to the PVDF membrane. After blocking with skim milk at room temperature for 1 h, the membrane was incubated overnight at 4°C with corresponding primary antibodies in a suitable dilution. The membranes were incubated with rabbit or mouse horseradish peroxidase‐conjugated secondary antibodies for 1 h at the room temperature and then detected using Luminata Crescendo Western HRP Substrate (Millipore, WBLUR0100).

### 2.11 Xenograft growth of PTC cells in mice

Four‐week‐old female BALB/c nude mice were purchased from Hunan Slack King of Laboratory Animal Co., Ltd. and maintained in specific pathogen‐free conditions. After randomly divided into different groups (five in each group), mice were injected subcutaneously with 0.1 ml of cell suspension containing 5 × 10^6^ cells. When a tumour was palpable, it was measured every 5 days and its volume was calculated according to the formula volume = 0.5 × length × width^2^. All experiments were performed in accordance with relevant institutional and national guidelines and regulations.

### 2.12 Immunohistochemistry (IHC)

Xenograft tumour tissue samples were fixed by formalin and dehydrated, and the 6‐mm‐thick sections were cut from paraffin‐embedded samples. The section was deparaffinized with xylene for 3 min (this process was repeated once), 100% ethanol for 5 min (this process was repeated once), 95% ethanol for 3 min and 70% ethanol for 2 min and washed with distilled water for 1 min. After antigen unmasking with citric antigen retrieval reagent (pH 6.0), immunoreactivity in sections was analysed by IHC using antibodies against Ki67 and PGK1, followed by counterstaining with haematoxylin, dehydration and mounting.

### 2.13 Statistical analysis

The results are shown as the mean SD and are representative of at least three independent experiments. Two‐tailed Student's *t* test, one‐way ANOVA and Mann–Whitney *U* test were performed to analyse differences between groups. *p* < 0.05 was considered statistically significant.

## 3 RESULTS

### 3.1 PTCSC3 is downregulated in PTC tissues

**FIGURE 1:**
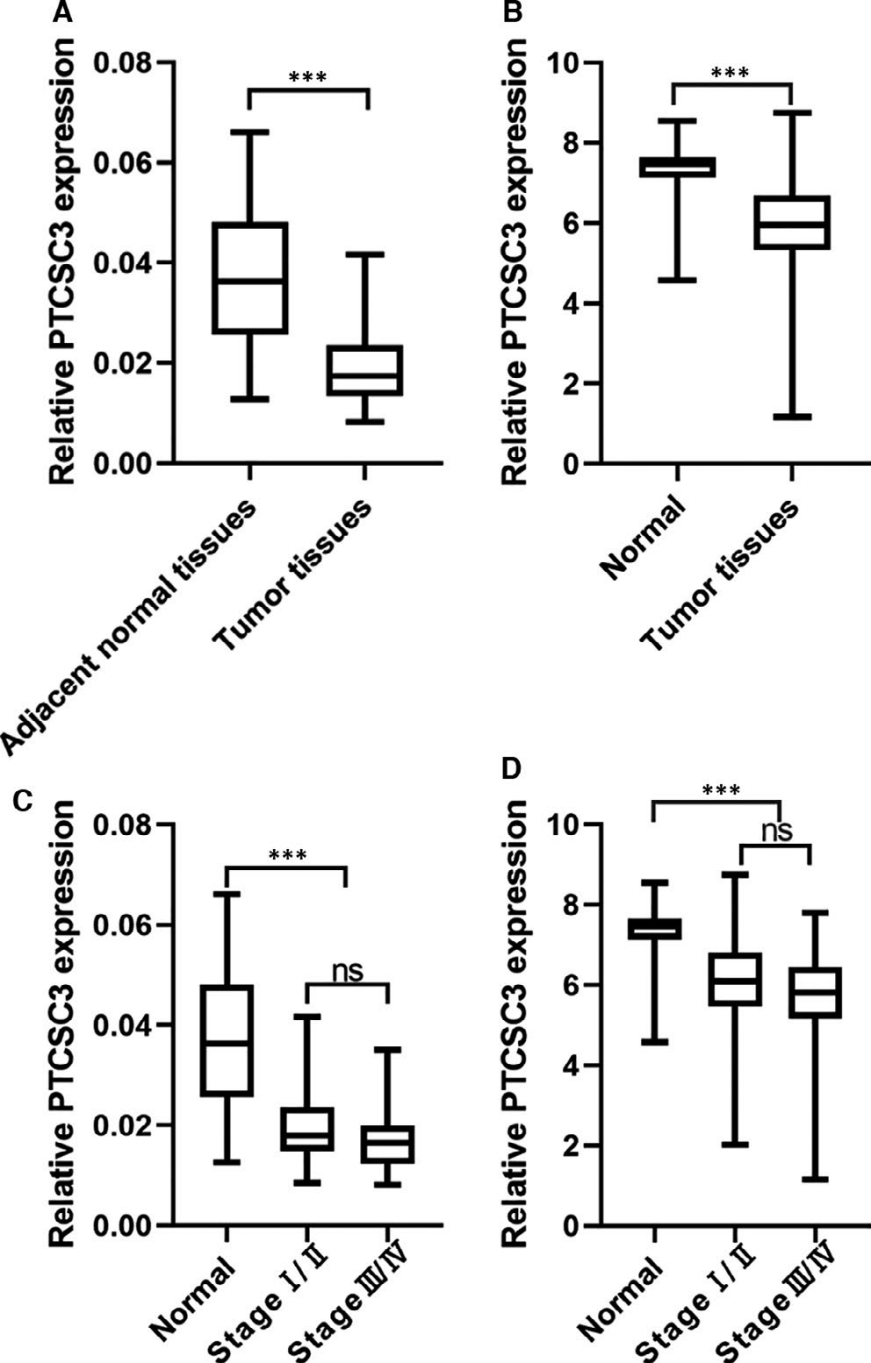
PTCSC3 is differentially expression in thyroid tumour and normal tissues. (A) Relative expression level of PTCSC3 in thyroid tumour tissues and their paired adjacent normal tissues from 68 patients. (B) TCGA database results showed that the relative expression level of PTCSC3 in thyroid tumour tissues (*n* = 467) was significantly lower than normal tissues (*n* = 59). (C) PTCSC3 expression in normal tissues (*n* = 68) and PTC clinical specimens at I/II stage (*n* = 40) and at III/IV stage (*n* = 28). ns, no significant difference; *p* < 0.01, Mann–Whitney *U* test. (D) PTCSC3 level in thyroid normal tissues and PTC clinical specimens at I/II stage and III/IV stage (TCGA)

To investigate the role of PTCSC3 in thyroid cancer, we analysed the expression of PTCSC3 in 68 pairs of frozen PTC samples and their adjacent normal tissues by using qRT‐PCR. The result confirmed that PTCSC3 expression level was substantially lower in PTC tissues than that in their adjacent tissues (Figure 1A). By using the Cancer Genome Atlas (TCGA) database analysis, we further demonstrated that PTCSC3 expression in PTCs was significantly downregulated than that in normal thyroid tissues (Figure 1B). Then, we assessed the clinical relevance of PTCSC3 and found that PTCSC3 expression levels were reduced significantly in all stages of thyroid cancer compared with the peritumoral tissues, but there was no significant difference in PTCSC3 expression between clinical stages Ⅰ/Ⅱ and Ⅲ/Ⅳ (Figure 1C,D), suggesting that PTCSC3 downregulation is an early event in PTC development.

### 3.2 PTCSC3 inhibit thyroid cancer cell proliferation in vitro and in vivo

**FIGURE 2:**
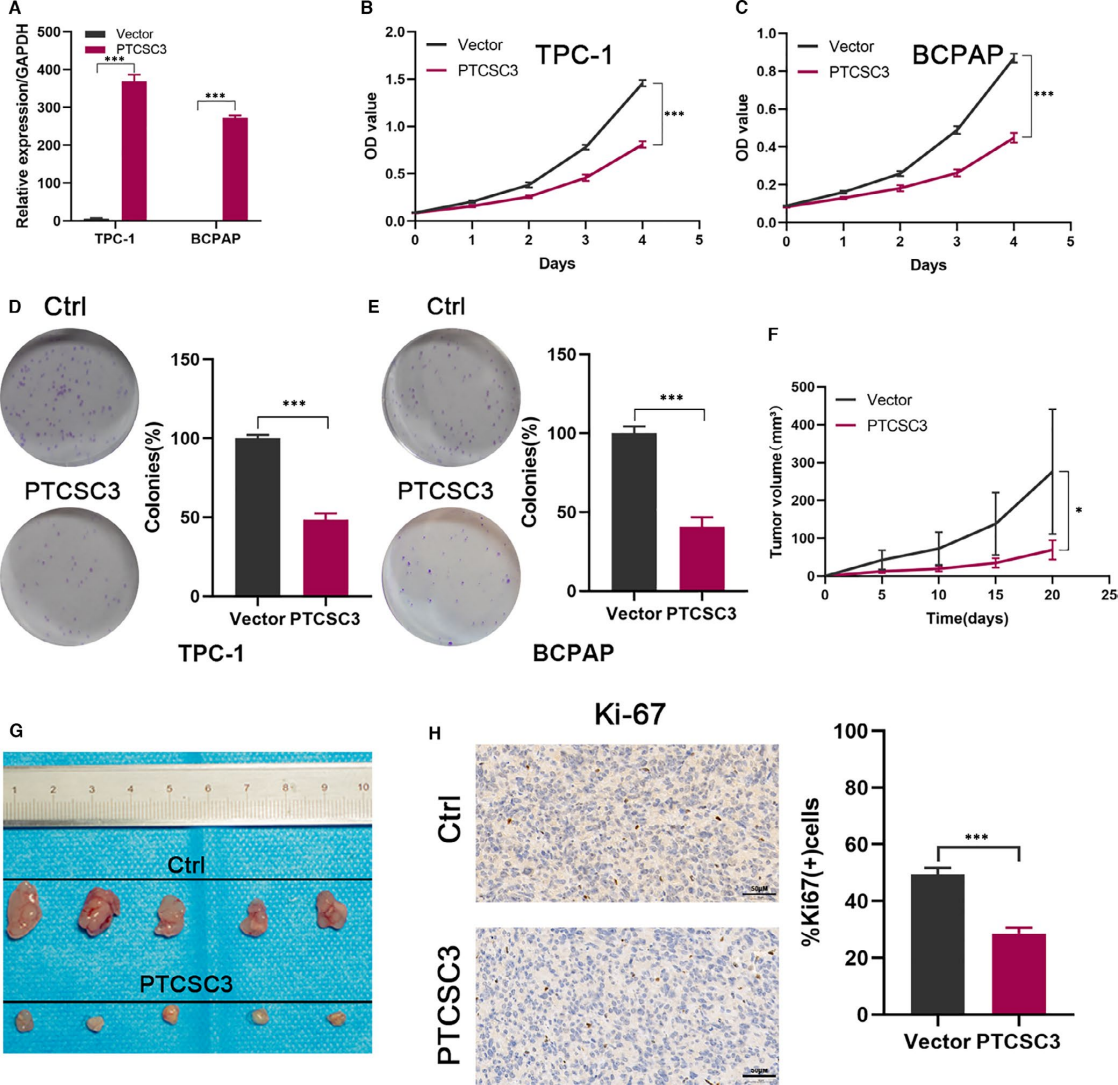
PTCSC3 overexpression suppressed thyroid cancer cells proliferation in vitro and vivo. (A) qRT‐PCR results showed PTCSC3 levels in PTCSC3/vector control transfected TPC‐1 and BCPAP cells. (B, C) the CCK8 assays show that overexpressed PTCSC3 in TPC‐1 and BCPAP resulted in suppressed proliferation capacity. (D, E) the colony formation ability was inhibited in PTCSC3 overexpression thyroid cancer cells. Left, the representative images of colony formation in the cells transfected with PTCSC3 or vector control. Quantitative analysis of colony numbers is shown on the right. (F) Tumour volume was measured every 5 day after the injection of PTCSC3 and control (*n* = 5) BCPAP cells’ groups (G) control and PTCSC3 overexpression BCPAP cells were injected into the nude mice. Tumours after removal from the mice. (H) Representative tumour sections from PTCSC3 overexpression and control mice were subjected to IHC staining using the indicated antibodies. *P < 0.05; ***P < 0.001

To elucidate the function of PTCSC3 in PTC, stable lentivirus‐infected TPC‐1 and BCPAP cell lines expressing PTCSC3 were established, which were validated at the RNA level. PTCSC3 expression was significantly increased in both TPC‐1 and BCPAP cells when compared with the control cells (Figure 2A). The CCK8 assay showed that increased expression of PTCSC3 substantially inhibited cell proliferation compared with the control group (Figure 2B,C). The inhibitory role of PTCSC3 on cell growth was further confirmed by colony formation assay. As shown in Figure 2D,E, PTCSC3 upregulation decreased the colony‐forming ability in both TPC‐1 and BCPAP cells. To further explore the impact of PTCSC3 on tumour growth in vivo, xenograft nude model was established. PTCSC3 upregulated and control BCPAP cells were injected subcutaneously into nude mice. As shown in Figure 2F,G, the tumours derived from PTCSC3 overexpressed cell line were significant smaller when compared with the control group. Moreover, the IHC results revealed that PTCSC3 overexpression remarkably decreased the expression levels of the cell proliferation marker Ki67 (Figure 2H). These results were consistent with our experimental results in vitro. Taken together, these data demonstrated that PTCSC3 upregulation suppresses thyroid cancer growth both in vitro and vivo.

### 3.3 PTCSC3 modulate glycolysis phenotype to regulate PTC proliferation

**FIGURE 3:**
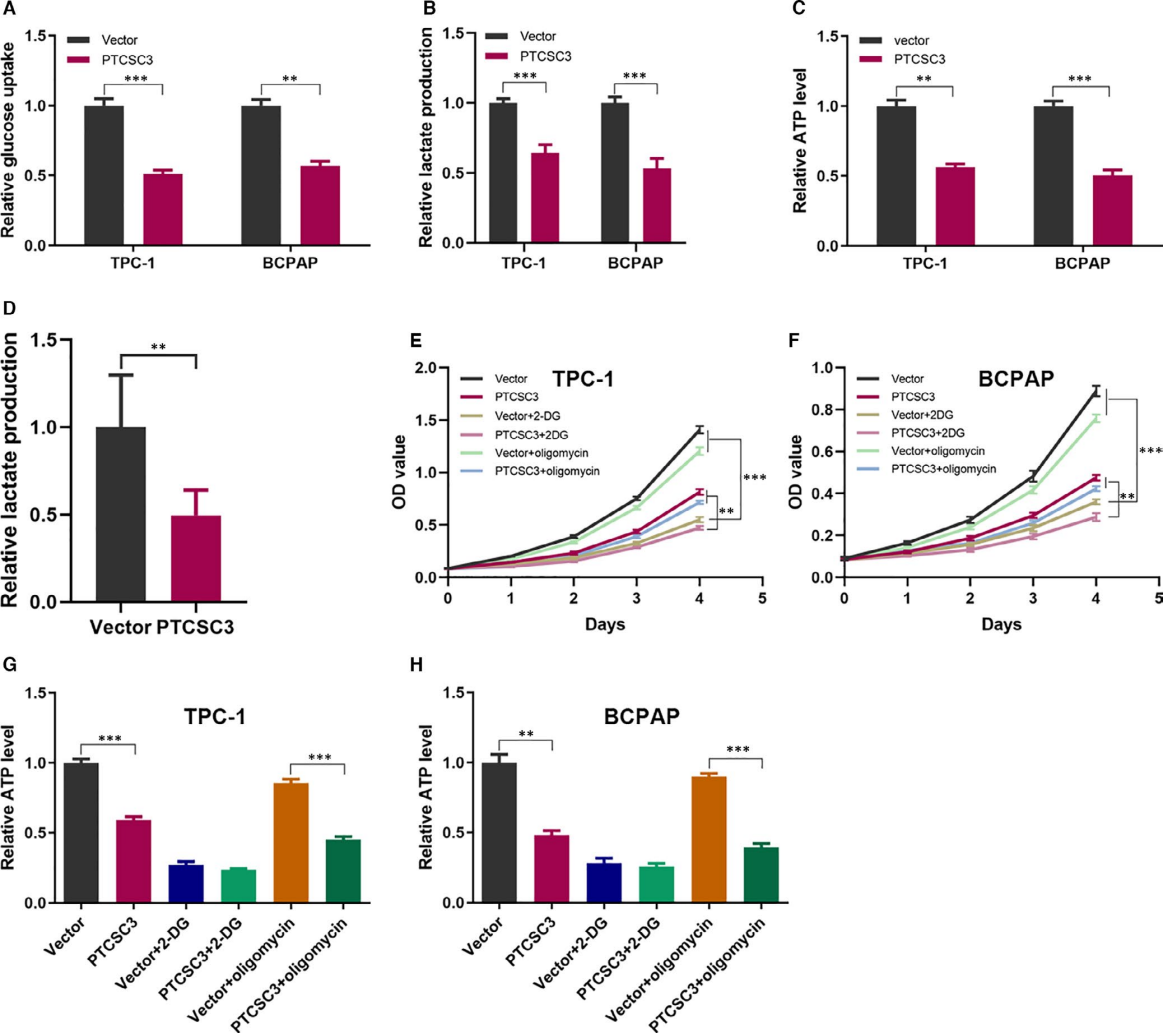
PTCSC3 inhibits thyroid cancer cell proliferation by suppressing glycolysis. (A) Relative glucose uptake in PTCSC3 overexpression TPC‐1 and BCPAP cells. (B) Relative lactate production in PTCSC3 overexpression TPC‐1 and BCPAP cells. (C) Relative ATP level in PTCSC3 overexpression TPC‐1 and BCPAP cells. (D) The lactate level of tumour tissue from PTCSC3 and control mice. (E, F) The proliferation curve of TPC‐1 and BCPAP cells transfected with PTCSC3 or vector control, treated with 2.5 mM 2‐DG or 0.1 mM oligomycin. (G, H) the relative ATP level of TPC‐1 and BCPAP cells transfected with PTCSC3 or vector control, treated with 2.5 mM 2‐DG or 0.1 mM oligomycin. **P < 0.01; ***P < 0.001

As tumour cells usually reprogramme their metabolism for rapid proliferation, in view of the role of PTCSC3 in the proliferation of thyroid cancer, we speculated that PTCSC3 was involved in the aerobic glycolysis regulation of PTC cells. To investigate whether PTCSC3 modulated glucose metabolism, the glucose uptake, lactate production and ATP level were measured. Expectedly, PTCSC3 led to a markedly decrease in glucose uptake, lactate production and ATP level in both TPC‐1 and BCPAP cell lines (Figure 3A–C), and the lactate levels in tumour tissues from control mice were substantially higher than those in PTCSC3 overexpression group (Figure 3D).

To further determine whether PTCSC3 reprogramming glycolysis devotes to its regulatory role in cell proliferation, we explored the role of PTCSC3 on cell proliferation after treatment with glycolytic inhibitors 2‐deoxy‐D‐glucose (2‐DG) and OXPHOS inhibitor oligomycin, respectively. The results demonstrated that 2‐DG obviously inhibited cancer cell proliferation, while oligomycin did not do so. Importantly, the difference in cell proliferation between the PTCSC3 overexpression cells and control cells was substantially reduced by 2‐DG treatment (Figure 3E,F). Consistent with the cell proliferation results, 2‐DG, but not oligomycin, significantly decreased the ATP production. Again, the difference in ATP production between the PTCSC3 overexpression cells and control cells was significantly decreased by 2‐DG treatment (Figure 3G,H). These data suggested that PTCSC3 inhibits PTC cell proliferation through suppressing glycolysis.

### 3.4 PTCSC3 physically interacts with PGK1 and promotes PGK1 degradation

**FIGURE 4:**
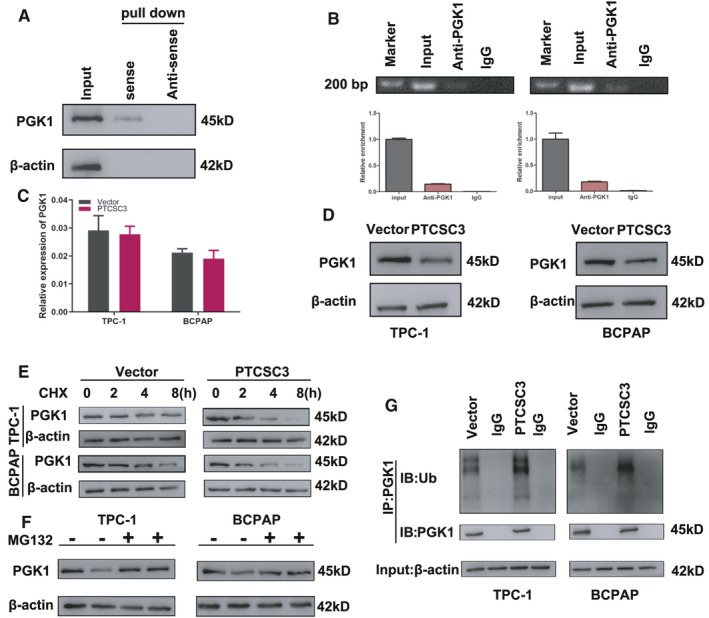
PTCSC3 interacts with and promotes PGK1 degradation via ubiquitination (A) Immunoblotting for specific associations of PGK1 with PTCSC3 from RNA pull‐down assays. (B) RIP assays show association of PGK1 with PTCSC3 in TPC‐1 and BCPAP overexpression cells. Relative enrichment represents RNA levels associated with PGK1 relative to an input control. Antibodies against PGK1 and control IgG served as controls. (C) Levels of PTPN11 mRNA were quantified by qPCR in TPC‐1 and BCPAP cells with stable overexpression of PTCSC3 or control. (D) Immunoblot analysis of PGK1 protein in TPC‐1 and BCPAP cells with stable overexpression of PTCSC3 or control. (E) TPC‐1 and BCPAP cells stably overexpressing PGK1 and control cells were treated with cycloheximide (CHX; 20 µg/ml) or vehicle for the indicated periods of time. PGK1 levels were analysed by immunoblotting. (F) TPC‐1 and BCPAP cells stably overexpressing PGK1 and control cells were treated with MG132 (10 µM) or vehicle for 12 h; immunoblotting for PGK1 levels in the indicated cells. (G) TPC‐1 and BCPAP cells with stable overexpression of PTCSC3 or control were treated with MG132 (10 µM) for 12 h. Cell lysates were immunoprecipitated (IP) with either control IgG or antibody against PGK1 and analysed by immunoblotting with ubiquitin and PGK1

It is well known that lncRNAs can drive many important cancer phenotypes through their interaction with protein. We proposed that PTCSC3 might interact with some proteins to perform its role in PTC development. To verify the hypothesis, RNA pull‐down assay followed by mass spectrometry analysis was conducted. Among the 54 identified potential PTCSC3‐interacting proteins (Table S1), since we focussed on glycolysis and we selected PGK1 for validation, our results confirmed that PGK1 directly interact with PTCSC3 (Figure 4A). Moreover, RNA immunoprecipitation (RIP) assays also demonstrated that PTCSC3 was substantially enriched in complexes precipitated with antibody against PGK1 compared with control IgG, determined by measuring co‐precipitated RNA by qPCR and RT‐PCR with agarose gel electrophoresis analysis (Figure 4B). The above results indicated that PGK1 specifically interact with PTCSC3.

To further clarify the molecular mechanism of the interaction between PTCSC3 and PGK1, we found that the levels of PGK1 mRNA were not altered (Figure 4C); however, the levels of PGK1 protein were dramatically reduced when PTCSC3 was overexpressed (Figure 4D). When treating PTCSC3 overexpressed TPC‐1 and BCPAP cells with the protein synthesis inhibitor cycloheximide, we found that the half‐life for PGK1 was markedly shorter than that in control cells (Figure 4E). Furthermore, when treating PTCSC3 overexpressed TPC‐1 and BCPAP cells with the proteasome inhibitor MG132, the protein levels of endogenous PGK1 increased more than those in control cells (Figure 4F), suggesting that PTCSC3 promotes the proteasome‐dependent degradation of PGK1 in thyroid cancer cells. Indeed, the ubiquitination of PGK1 was dramatically increased in cells overexpressing PTCSC3 in comparison to control cells (Figure 4G). Taken together, these results implicated that the interaction of PTCSC3 with PGK1 promotes degradation of PGK1 through ubiquitination.

### 3.5 PTCSC3 suppresses proliferation and the Warburg effect via PGK1

**FIGURE 5:**
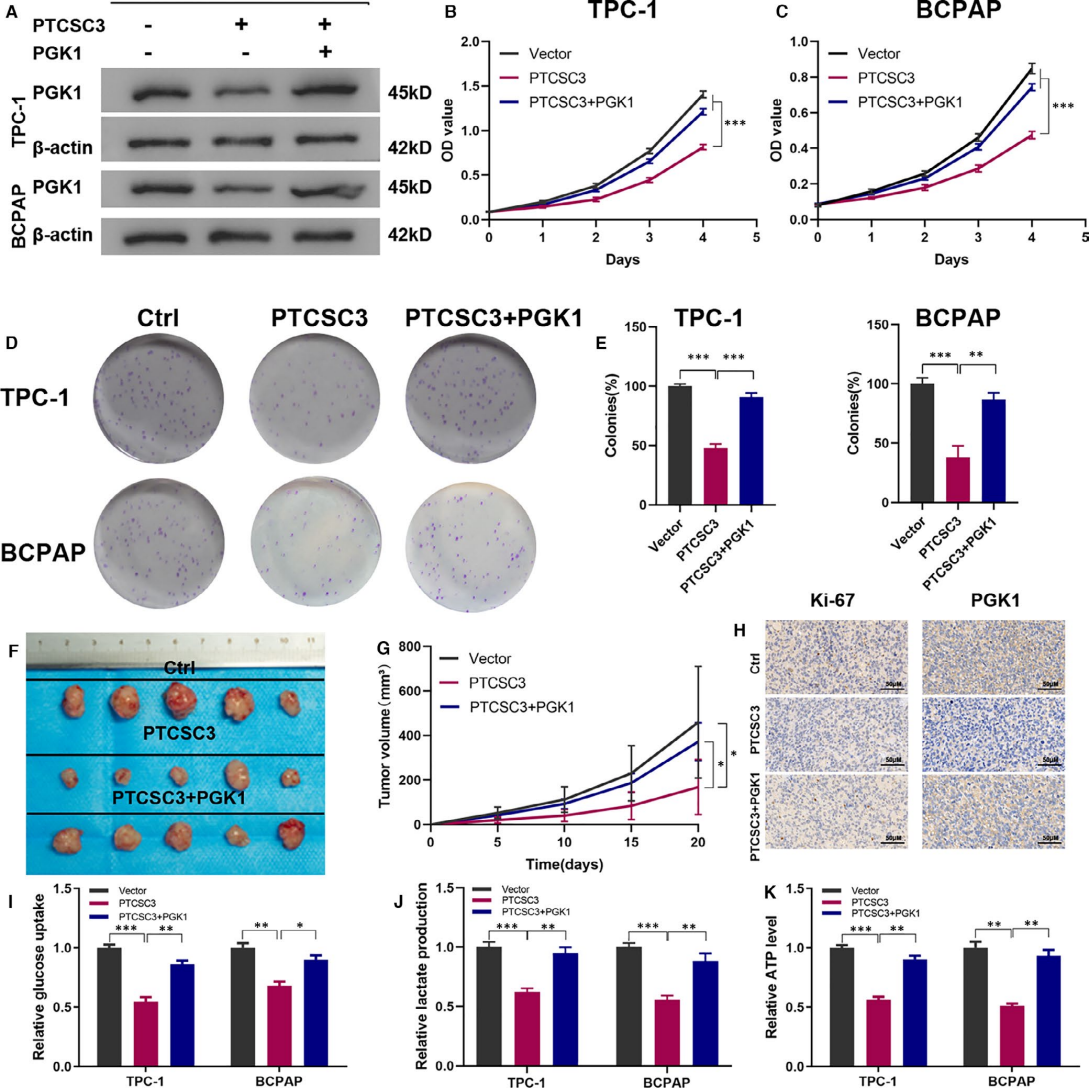
PTCSC3 inhibits proliferation and glycolysis partially via PGK1. (A) Overexpression efficiency of PGK1 in PTCSC3 overexpression TPC‐1 and BCPAP cells analysed by immunoblotting. (B, C) PGK1 overexpression decreased the inhibitory effects of the overexpression of PTCSC3 on the CCK8 assay of TPC‐1 and BCPAP cells. (D, E) PGK1 overexpression attenuated the inhibitory effects of the overexpression of PTCSC3 on the colony formation properties of TPC‐1 and BCPAP cells. (F) Photographs of dissected tumours from nude mice were presented. (G) Tumour growth curves were measured every 5 day among vector control, overexpressed PTCSC3 and PGK1 overexpression after overexpressed PTCSC3 cells in nude mice. (H) Respective IHC staining of Ki67 and PGK1 in orthotopic xenograft model. (I–K) Relative glucose uptake, lactate production and ATP level were determined in the different groups. *P < 0.05; **P < 0.01; ***P < 0.001

Our results demonstrated that PTCSC3 directly binds to PGK1, suggesting that the interaction between PTCSC3 and PGK1 might contribute to the development of PTC. When PGK1 was overexpressed (Figure S1A), the proliferation abilities of TPC‐1 and BPCPAP cells were remarkably increased (Figure S1B–D). Unsurprisingly, in the presence of PGK1 overexpression, the glucose uptake, lactate production and ATP level were significantly increased (Figure S1E–G). To further investigate whether PTCSC3 performs an anti‐tumour function via PGK1, we overexpressed PGK1 in PTCSC3‐overexpressing TPC‐1 and BCPAP cells, and their protein expression was confirmed by Western blot (Figure 5A). The proliferation inhibitory effect of PTCSC3 was significantly attenuated by PGK1 overexpression, demonstrating by CCK8 and colony formation assays (Figure 5B–E). To further confirm the tumour‐suppressive effect of PTCSC3 via PGK1 in vivo, the orthotopic xenograft model was established. As shown in Figure 5F,G, PGK1 overexpression impaired the inhibitory effect of PTCSC3 in tumour progression. Same results were observed with IHC staining (Figure 5H). In addition, as revealed by glucose consumption, lactate production and ATP level assays (Figure 5I–K), overexpression of PGK1 rescued the inhibitory effect of glycolysis by PTCSC3.

## 4 DISCUSSION

Although the pathogenesis of papillary thyroid carcinoma has been extensively investigated, the key molecular mechanism of its initiation and development remains obscure. Thus, improved knowledge of molecular mechanism underlying the thyroid cancer is necessary to ensure that cancer patients could be provided with more effective treatment opinions.

Recently, increasing evidences have revealed that lncRNAs are involved in tumour initiation and development and are clinically useful in diagnosis and prognosis. Though some lncRNAs were found to be functional in PTC. Such as LncRNA AB074169 functions as a tumour suppressor during PTC tumorigenesis via modulation of KHSRP‐mediated CDKN1a expression.^32^ However, the involvement of LncRNAs in PTC progression is still elusive. In this study, we found that PTCSC3 expression is substantially decreased in PTC tissues compared with adjacent tissues, whereas there was no difference of PTCSC3 expression among different stages, suggesting that PTCSC3 contributes to the development of PTC. The experimental data in our study proved that PTCSC3 functions as a tumour suppressor, overexpression of PTCSC3 could inhibit PTC cell proliferation and development in vitro and in vivo.

To date, it has been fundamentally recognized that the atypical metabolic phenomenon is common for virtually all types of cancer.^10^ The abnormal metabolic through increasing glycolysis provided cancer cells more biosynthetic precursors for rapid macromolecule synthesis, and advantages to maintain cellular redox homeostasis for better survival.^33^ Our results proved that PTCSC3 can reprogramme glucose metabolism. Moreover, we found that the differences in proliferation and ATP production by PTCSC3 were almost eliminated after treatment with 2‐DG, revealing that PTCSC3 plays a role in development via coordinate glycolysis in further.

It has been shown in previous study that LncRNA exerts its biological function mainly by binding to RBP. In this study, we performed RNA pull‐down assay and mass spectrometry to identify specific association of PTCSC3 with PGK1, which was further confirmed by Western blotting and RIP. PGK1, the central enzyme in the glycolysis pathway, controls the production of ATP during aerobic glycolysis and is upregulated in many types of human cancers.^34^ As the nutrients, oxygen and signalling stimulus dynamically changing, cells have to adapt to the varied environment to growth and proliferation. Through the structure and function of many proteins regulated by post‐translational modification (PTM) in response to certain stimuli, it makes cells effectively regulate metabolism to survive and thrive in the dynamically changing microenvironment.^35^ There were mounting evidences showing that PGK1 was regulated by different PTMs. For example, O‐GlcNAcylation of PGK1 at threonine 255 regulates the glycolysis and promotes the development of colon cancer,^36^ acetylation of PGK1 at K323 contributes to liver progression,^13^ phosphorylation of PGK1 at Y324 devote to glioblastoma multiforme formation.^37^ Here, we show that PGK1 was post‐translationally regulated by PTCSC3 via facilitating its ubiquitin‐mediated degradation in PTC. However, it remains to be investigated whether PTCSC3 promotes the ubiquitination of PGK1 by directly changing the conformational structure which leads to the exposure of the recognition domain of ubiquitin proteins or serving as a scaffold to provide a platform for PGK1 interacting with the certain protein.

In conclusion, our data demonstrate that PTCSC3 is a critical suppressor in PTC which can inhibit PTC cell proliferation through inhibiting glycolysis and promoting the ubiquitin‐mediated degradation of PGK1. From a standpoint of controlling the initiation and development of PTC, the study provided new insight into mechanism of PTC. As such, PTCSC3 can be regarded as the key mediator and promising target for controlling the initiation and development of PTC.

## CONFLICT OF INTEREST

The authors declare that they have no conflict of interest.

## AUTHOR CONTRIBUTION

**Bo Jiang:** Investigation (equal); Methodology (equal); Validation (lead); Writing‐original draft (lead). **Yong Chen:** Methodology (equal); Validation (supporting). **Fada Xia:** Formal analysis (equal); Software (equal); Writing‐original draft (supporting). **Xinying Li:** Writing‐review & editing (lead).

## Supporting information

Fig S1

Table S1

## Data Availability

The data that support the findings of this study are available from the corresponding author upon reasonable request.
