# Supplementary figures and images for: PTCSC3‐mediated glycolysis suppresses thyroid cancer progression via interfering with PGK1 degradation
Source: J Cell Mol Med. 2021 Aug 1;25(17):8454–63. doi: 10.1111/jcmm.16806 (PMC8419167; doi:10.1111/jcmm.16806)

**A**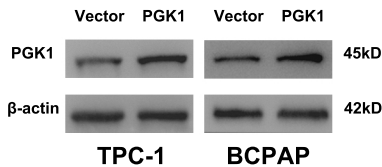**B**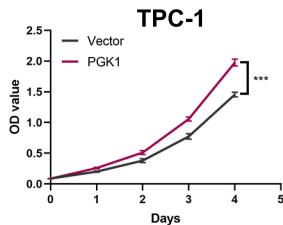**C**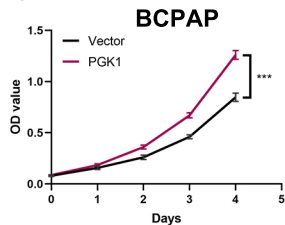**D**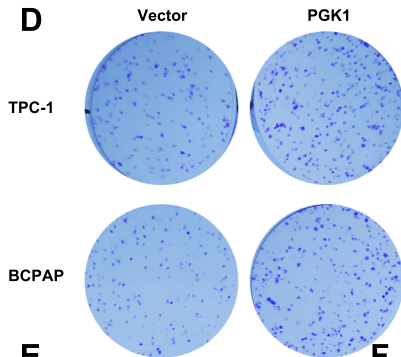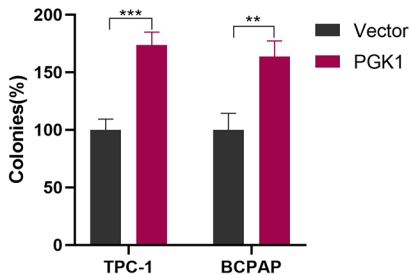**E**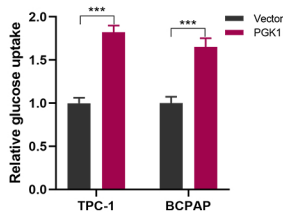**F**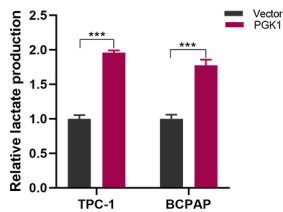**G**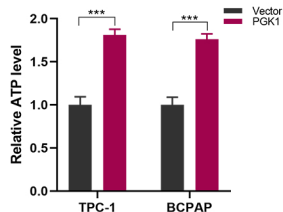

Supplement: Supplementary file 1 — Fig S1 [file JCMM-25-8454-s001.pdf]
