# Supplementary material for: PTCSC3‐mediated glycolysis suppresses thyroid cancer progression via interfering with PGK1 degradation
Source: J Cell Mol Med. 2021 Aug 1;25(17):8454–63. doi: 10.1111/jcmm.16806 (PMC8419167; doi:10.1111/jcmm.16806)
Supplement: Supplementary file 2 — Table S1 [file JCMM-25-8454-s002.docx]

| Supplement Table 1. Proteins Identified In LS-MS | | |
| --- | --- | --- |
| Protein Name | UniProtKB AC | Percent Coverage |
| TPM2 | P07951 | 38 |
| ACTA2 | P62736 | 28 |
| PABPN1 | Q86U42 | 59 |
| ACTN1 | P12814 | 13 |
| ARPC5L | Q9BPX5 | 78 |
| PGK1 | P00558 | 77 |
| MELTF | P08582 | 14 |
| PIP | P12273 | 10 |
| MROH1 | Q8NDA8 | 64 |
| FOSL2 | P15408 | 52 |
| WDR1 | O75083 | 13 |
| SNU13 | P55769 | 94 |
| ITPR1 | Q14643 | 40 |
| LARGE2 | Q8N3Y3 | 18 |
| MYZAP | P0CAP1 | 30 |
| IFIT2 | P09913 | 36 |
| KBTBD3 | Q8NAB2 | 12 |
| PPIA | P62937 | 73 |
| VCAM1 | P19320 | 18 |
| PPEF2 | O14830 | 16 |
| SMC1B | Q8NDV3 | 80 |
| HBB | P68871 | 88 |
| PPIL2 | Q13356 | 27 |
| SIPA1L1 | O43166 | 70 |
| CRYZL1 | O95825 | 43 |
| TTC5 | Q8N0Z6 | 20 |
| STARD13 | Q9Y3M8 | 69 |
| FEZ1 | Q99689 | 18 |
| RPL29 | P47914 | 94 |
| RUNX1T1 | Q06455 | 17 |
| ZNF217 | O75362 | 10 |
| BST1 | Q10588 | 25 |
| SIPA1L3 | O60292 | 60 |
| CLPB | Q9H078 | 10 |
| POLDIP2 | Q9Y2S7 | 35 |
| MINDY2 | Q8NBR6 | 16 |
| EXOSC6 | Q5RKV6 | 59 |
| LOXL3 | P58215 | 16 |
| GEMIN5 | Q8TEQ6 | 69 |
| MS4A10 | Q96PG2 | 34 |
| CCT7 | Q99832 | 24 |
| BRCC3 | P46736 | 28 |
| WDR92 | Q96MX6 | 34 |
| KIF1B | O60333 | 70 |
| CTNNA2 | P26232 | 51 |
| SNRPB | P14678 | 33 |
| RPS24 | P62847 | 90 |
| BCL2L14 | Q9BZR8 | 67 |
| SNRPD3 | P62318 | 71 |
| ABCA2 | Q9BZC7 | 70 |
| NDUFV2 | P19404 | 52 |
| ZNF681 | Q96N22 | 25 |
| SEPT14 | Q6ZU15 | 21 |
